# Supplementary material for: Beehive scale-free emergent dynamics
Source: Sci Rep. 2024 Jun 11;14:13404. doi: 10.1038/s41598-024-64219-w (PMC11167022; doi:10.1038/s41598-024-64219-w)
Supplement: Supplementary file 1 — Supplementary Information. [file 41598_2024_64219_MOESM1_ESM.pdf]

Supplementary materials for the paper "Beehive scale-free emergent dynamic"

The supplementary materials include additional information concerning the figures presented in the main body of the paper. S1 depicts the relationships between various metrics presented in Figure 1. Additionally, it presents the results for the computation of root mean square displacement (RMSD) computed every hour for all bees. This metric (1) quantifies the displacement of particles with respect to the reference position $\vec{r}(0)$.

$$RMSD = \langle \sqrt{(\vec{r}(0) - \vec{r_t})^2} \rangle \tag{1}$$

RMSD exponent a is obtained by solving the relationship $\log_{10}(RMSD) \propto a \log_{10}(t)$. Our analysis shows that the values of the exponent have a mean of 0.66 which is close to the theoretical value of $RMSD \propto t^{2/3}$ for Brownian motion in 2D.

S2 expands the analysis of traffic presented in the Figure 4. In addition to computing the relationship between occupancy and traffic, we compute a metric known as burstiness B, to characterize the nature of traffic flow at different values of occupancy. We use the same grid partition and intervals as for the computation of the results presented in Figure 4. For each interval, we consider inter-event distribution τ comprised of the times elapsed between occurrences of bee crossing the midline of the square.

$$B = \frac{\sigma_\tau - \mu_\tau}{\sigma_\tau + \mu_\tau} \tag{2}$$

B is computed by considering the relationship between the μ_τ and σ_τ. This metric ranges from -1 to 1, and its minimal value corresponds to a completely regular signal. Results presented in 2 indicate that the bee traffic has a bursty nature, furthermore traffic at high occupancy values is characterised by enhanced burstiness. This observation is particularly pronounced when intervals with high occupancy which correspond to Phase I (brown dots) are considered.

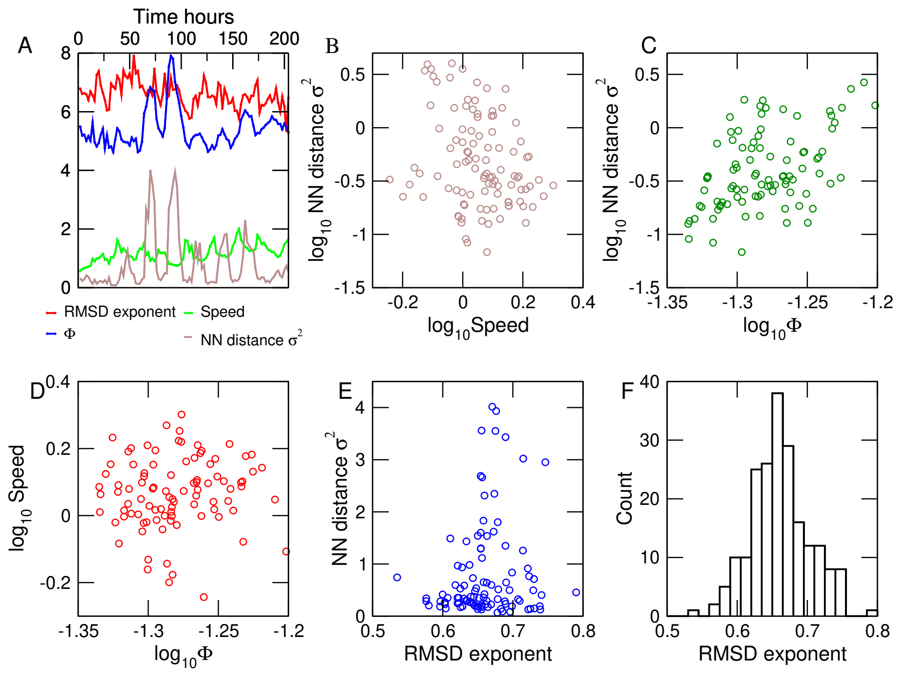

S 1: Relationship between different metrics. Pane A depicts the timeseries of mean speed, variance σ^2 of the distance between nearest neighbors, polarisation Φ and the exponent of the RMSD. For visual clarity values of of Φ

and the RMSD exponent have been multiplied by 100 and 10 respectively. Average values computed every two hours are presented for all the metrics. Pane B depicts the relationship between the mean speed and variance of the NN distance in the logarithmic coordinates. Pane C presents the relationship between polarization and NN distance σ^2. Pane D presents the relationship between the Φ and mean speed. Pane E depicts the relationship between the RMSD exponent and variance of NN distance. Pane F presents the distribution of the RMSD displacement exponent computed every hour.

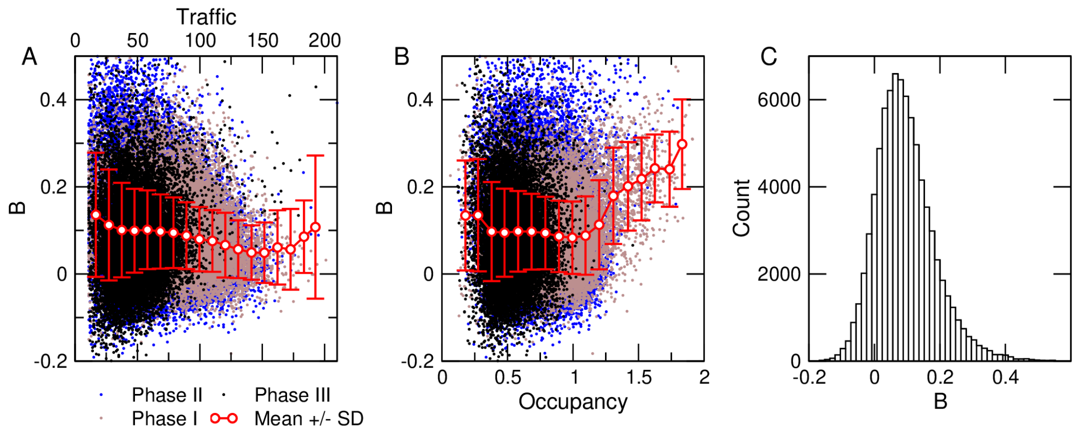

S 2: Burstiness metric. The color distinguishes different phases of the experiment: brown (phase I) before the hive was open, black (phase III), after foragers have been removed from the hive and blue the remaining part (phase II). The red line with white circles represents the binned average (+/- SD) computed for all the points irrespective of the phase. Pane A: Burstiness as a function of traffic. Pane B: Burstiness as a function of occupancy. Pane C: Histogram of burstiness for all phases of the experiment.

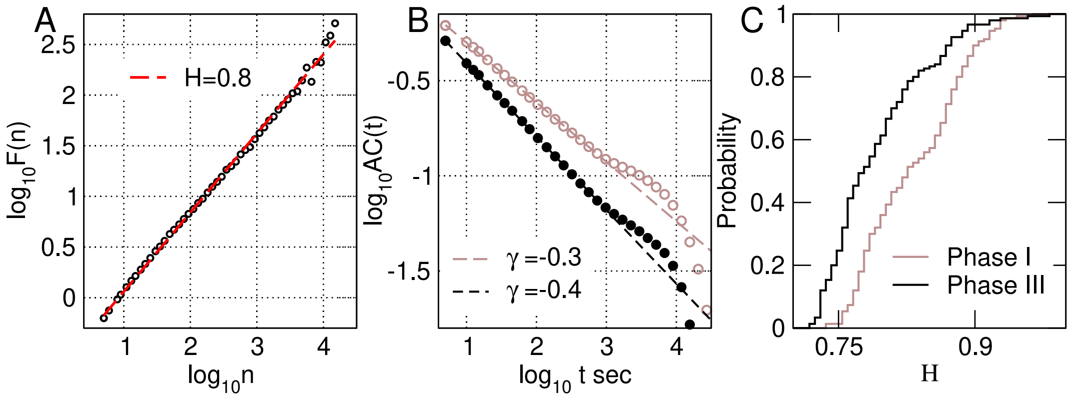

S 3: Detrended fluctuation analysis and decay of temporal correlations. Pane A presents the scaling of fluctuations $F(n)$ as a function of n, computed for a time series of occupancy fluctuation of a single randomly chosen grid site for the duration of phase I. Note that the scaling extends more than 3 orders of magnitude in n. Pane B presents the decay of the auto-correlation function AC as a function of t for Phases I (brown circles) and III (filled black circles). Pane C shows cumulative distribution of Hurst exponents H for timeseries of occupancy fluctuation of all grid sites from Phase I(brown) and Phase III(black). Mean H values for phases I and II are $\langle H_1 \rangle = 0.84$ and $\langle H_3 \rangle = 0.79$ respecetively.

Figure S3 elucidates the behavior of the autocorrelation in the fluctuations of occupancy. The same coarse-graining is used as in Figure 4 in the main body of the paper. We compute fluctuation scaling with time for all time series of occupancy fluctuations at individual grid sites using detrended fluctuation analysis(DFA). An illustrative example is presented in pane A. Note that the scaling exponent $H \propto 0.8$ indicates the presence of long-range correlations in the time-series. Distribution of H for all grid sites shown in pane C indicates that on average H values are larger during the Phase I. Additionally, we compute the decay of auto-correlation function AC as a function of time t. Auto-correlation functions are computed for all gridsites and their log-binned average is used to obtain the decay function presented in pane B. Note that in both cases auto-correlation decays with time as power-law, $AC(t) \propto t^{\gamma}$, which is consistent with the $H > 0.5$. For Phase III, when the density is lower, decay happens faster ($\gamma \propto 0.4$) as compared to the Phase I($\gamma \propto\ 0.3$).

Timeseries with long range dependencies are characterized by a number of key exponenets: the slope of the auto-correlation function γ, the Hurst exponent H and the spectral scaling exponent β. The latter describes the scaling of the power spectra of the timeseries S as a function of frequency f, $S(f) \propto f^{-\beta}$. These exponents are connected by a fundamental scaling relationships (3).

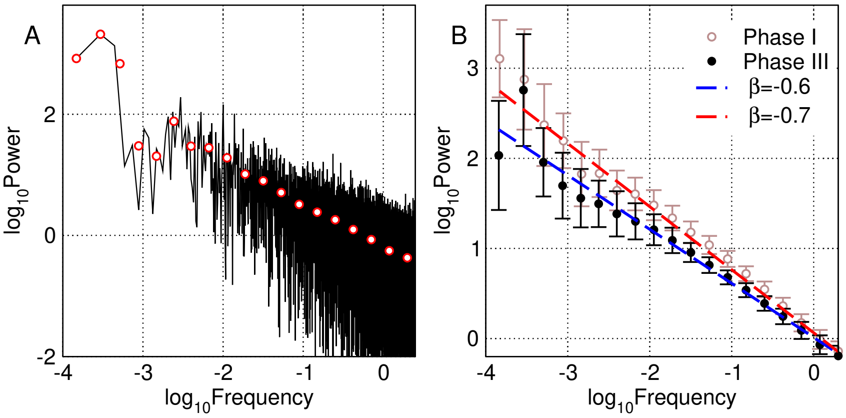

S 4: Spectral analysis of occupancy fluctuations time-series. Pane A depicts illustrative results for **one** of the grid sites for the duration of the Phase I. Red circles show logarithmic binning of the data. Pane B shows statistical averages of the power spectra computed for all grid sites of the Phase I(hollow brown circles) and Phase III (filled black circles). Dashed red and blue lines show theoretically expected scaling of $S(f) \propto f^{-\beta}$ with $\beta = 1 - \gamma$ computed using the values of the auto-correlation exponents γ from figure S 3 for both phases.

$$\begin{aligned} \beta &= 1 - \gamma \\ \beta &= 2H - 1 \\ \gamma &= 2 - 2H \end{aligned} \tag{3}$$

To elucidate these relationship in our data we compute the power spectra of occupancy fluctuations at individual grid sites, pane A in the S4 shows an illustrative example while pane B presents log-binned averages for all grid sites. It can be observed that for lower frequencies average value closely align with theoretical scaling. Furthermore both β values lie within the range of $0.5 < \beta < 1.5$ which is indicative of the processes with long range temporal dependencies. The remaining scaling relationship are also fulfilled within reasonable margin of error when statistical averages are considered. In all cases Phase I is characterized by ehcanced temporal dependencies as compared to phase III. These observation provide additional confirmation, that correlations in the bee-hive are dependent on its density.

I. SUPPLEMENTARY TABLES

Tables I and II present supporting information to Figures 4 and 5 in the main body of the paper. Table I presents the goodness of fit metrics for the exponents fitted to the linear regions of distance-decay functions for correlations of occupancy fluctuations (Figure 4A), speed (Figure 5) as well as for relation between $1 - AC(1)$ and the number of time-series (Figure 4B). In all cases, exponent values have been obtained by solving the linear regression on log-transformed data, using the least-squares method. We note, that as is demonstrated in Table I, the residuals are normally distributed in all cases, therefore solution obtained through linear regression is equivalent to the one derived using the MLE (Maximum Likelihood estimation).

Table II summarizes the results of an additional computation which entertains the possibility that the distance-decay of correlations can be interpreted as the exponential decay $C(r) = ae^{-\lambda r}$ as compared to the hypothesis that correlations decay as power-law $C(r) = br^{-a}$ with distance. Alternative fits are compared by computing the MSE (Mean Squared error) and AIK (Akaike information criterion). Note, that except for the decay of correlations in speed in Phase III, both metrics indicate that the power-law describes the decay of correlation better than the exponential function. Such observation is consistent with the hypothesis that the bee hive exhibits a density-dependent jamming transition, stronger correlations occur at critical density and decline, once the density is reduced.

**Phase**	a	R^2	KS statistic
Figure 4A			
Phase I	-0.5	0.98	0.15
Phase II	-0.3	0.99	0.25
Phase III	-0.75	0.98	0.19
Figure 4B			
Phase I	-0.84	0.99	0.25
Phase II	-1.015	0.99	0.26
Phase III	-0.7	0.99	0.19
Figure 5			
Phase I	-0.2	0.91	0.21
Phase II	-0.59	0.96	0.15
Phase III	-0.93	0.83	0.2

TABLE I: This table provides additional information concerning the linear fits in Figures 4A, 4B, and Figure 5. The first column indicates the phase of the experiment, second and third columns show the exponents and the corresponding R^2 values. The last column presents the KS statistic, obtained by comparing the distribution of residuals to the normal distribution using the one-way Kolmogorov–Smirnov test. P-values for these tests have been in all cases significantly higher than alpha=0.05, thereby supporting the hypothesis that residuals are distributed normally. Note that fit for the speed in Phase 3 is provided in the table, but not in the main figure, as its quality was deemed insufficient.

**Phase**	$\ln MSE_1$	$\ln MSE_2$	AIC_1	AIC_2
Figure 4A				
Phase I	-11.31	-14.35	-165.6	**-211.3**
Phase II	-13.45	-16.67	-197.8	**-246.0**
Phase III	-13.97	-14.57	-191.5	**-199.9**
Figure 4B				
Phase I	-14.58	-15.64	-68.9	**-74.2**
Phase II	-13.88	-15.52	-65.4	**-73.6**
Phase III	-14.82	-15.3	-55.3	**-57.2**
Figure 5				
Phase I	-8.0	-9.47	-92	**-109.6**
Phase II	-7.86	-9.21	-74.6	**-88.1**
Phase III	-11.57	-8.43	**-88.5**	-63.4

TABLE II: The table summarizes the comparison between the power-law and exponential fits for the distance decay of correlations. Subscript 1 indicates the exponential fit and subscript 2 the power-law. The first two columns $\ln MSE_1$ and $\ln MSE_2$ show the natural logarithm of the Mean squared error for the power-law and exponential decay respectively, while the last two columns present the scores of Akaike information criterion, with lower values indicated with bold text.
